# Moving the mountain: analysis of the effort required to transform comparative anatomy into computable anatomy

**DOI:** 10.1093/database/bav040

**Published:** 2015-05-13

**Authors:** Wasila Dahdul, T. Alexander Dececchi, Nizar Ibrahim, Hilmar Lapp, Paula Mabee

**Affiliations:** ^1^Department of Biology, University of South Dakota, Vermillion, SD, USA, ^2^Department of Organismal Biology and Anatomy, University of Chicago, Chicago, IL, USA and ^3^National Evolutionary Synthesis Center, Durham, NC, USA

## Abstract

The diverse phenotypes of living organisms have been described for centuries, and though they may be digitized, they are not readily available in a computable form. Using over 100 morphological studies, the Phenoscape project has demonstrated that by annotating characters with community ontology terms, links between novel species anatomy and the genes that may underlie them can be made. But given the enormity of the legacy literature, how can this largely unexploited wealth of descriptive data be rendered amenable to large-scale computation? To identify the bottlenecks, we quantified the time involved in the major aspects of phenotype curation as we annotated characters from the vertebrate phylogenetic systematics literature. This involves attaching fully computable logical expressions consisting of ontology terms to the descriptions in character-by-taxon matrices. The workflow consists of: (i) data preparation, (ii) phenotype annotation, (iii) ontology development and (iv) curation team discussions and software development feedback. Our results showed that the completion of this work required two person-years by a team of two post-docs, a lead data curator, and students. Manual data preparation required close to 13% of the effort. This part in particular could be reduced substantially with better community data practices, such as depositing fully populated matrices in public repositories. Phenotype annotation required ∼40% of the effort. We are working to make this more efficient with Natural Language Processing tools. Ontology development (40%), however, remains a highly manual task requiring domain (anatomical) expertise and use of specialized software. The large overhead required for data preparation and ontology development contributed to a low annotation rate of approximately two characters per hour, compared with 14 characters per hour when activity was restricted to character annotation. Unlocking the potential of the vast stores of morphological descriptions requires better tools for efficiently processing natural language, and better community practices towards a born-digital morphology.

**Database URL:** http://kb.phenoscape.org

## Introduction

The conversion of text-based descriptions of phenotypes from the descriptive and phylogenetic literature into a computable format is a critical component in linking phenotypic data to genes and the environment. Unfortunately, to date there is no straightforward and scalable means to automatically transform the legacy literature into a computable, i.e. semantic, framework (1). Over the past 8 years, the Phenoscape project (2) has striven to overcome these challenges and to convert phenotypes from the evolutionary biology literature into a semantic representation to prototype the connection between species phenotypes and model organism genotypes. The resulting linked data in the Phenoscape Knowledgebase (kb.phenoscape.org) are a rich resource of evo-devo hypotheses and inferred phenotypic data (2). This wealth of annotations, however, represents a small fraction of the phenomic richness of extinct and extant life recorded in the literature. To assess the means required to scale up this approach, we undertook an analysis of the time and resources that were required for two Phenoscape studies that involved significant annotation.

**Figure 1.**
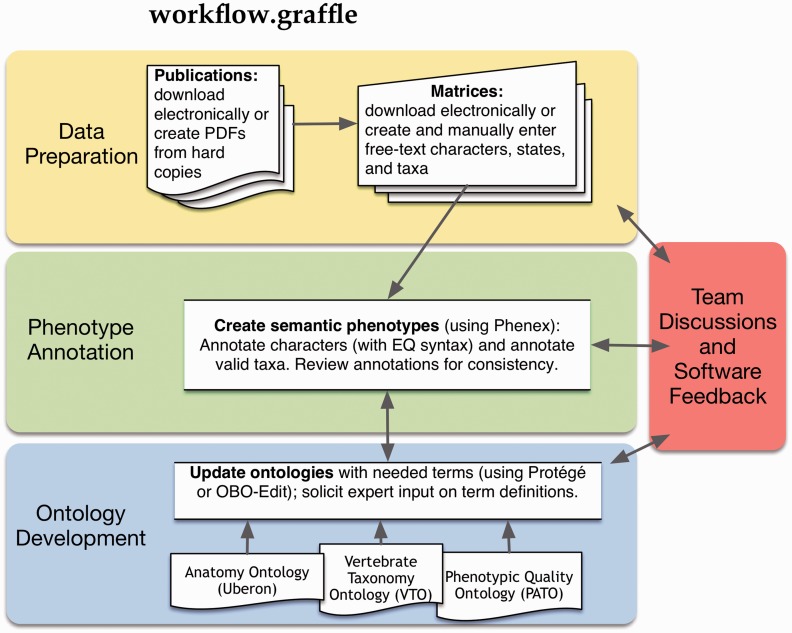
Workflow for the curation of phenotypic characters from systematic studies.

The annotation of species phenotypes involves attaching fully computable logical expressions following the Entity-Quality (EQ) formalism (3) to free-text. The EQ method was originally developed by the model organism community to represent gene phenotypes using ontology terms. By virtue of axioms in the requisite ontologies, ontology terms are logically related to one another, and thus collections of EQ descriptions, herein termed ‘phenotypes’ can be queried and reasoned across using a number of relationships, including subsumption and parthood relations. For example, the anatomical description ‘shape of cleithrum: triangular’ is represented as Entity (E): ‘cleithrum’ (a term from an anatomy ontology) and Quality (Q): ‘triangular’ (a term from a quality ontology). The cleithrum is a dermally derived bone and part of the pectoral girdle skeleton. Thus a query on E: ‘dermal bone’ will return the above annotation to E: ‘cleithrum’, along with other phenotypes annotated to types of dermal bones (e.g. E: ‘frontal bone’). Similarly a search for parts of the ‘pectoral girdle skeleton’ would return the annotation to E: ‘cleithrum’ along with phenotypes annotated to other entities of this region such as ‘scapula’. To facilitate an efficient data curation workflow (Figure 1), we found it necessary to develop specialized software tools, in particular Phenex (4) for the annotation of phylogenetic matrix-based phenotypes from the evolutionary literature. Phenex was continuously updated and improved during the course of manual curation (5) to streamline the curation process and workflow, and to improve the allocation of curators’ time. For example, our previous work indicated that shifting curators’ attention from annotation to ontology development was a major bottleneck in the annotation workflow (5), due to the associated switch of context and software tools. To address this, we developed a feature in Phenex that decouples annotation from ontology development (6), enabling the curator to continue focusing on phenotype annotation even when required terms were missing from the requisite ontologies. Here, we describe and quantify the discrete tasks involved in translating free-text into a computable format and assess which limitations to curation speed and scalability are imposed by the nature of this work and where additional gains in efficiency can be made to facilitate scaling up phenotypic curation to encompass the full morphological and phylogenetic literature. Quantifying curation processes and documenting the issues faced are necessary for funding agencies, reviewers and researchers to understand what goes into the resources they support and use.

## Methods

**Figure 2.**
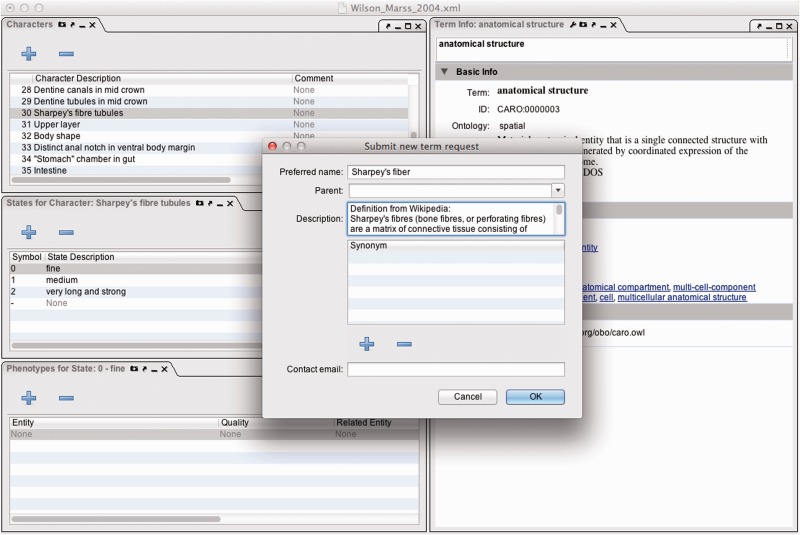
Phenex screenshot of window with the ontology request broker (ORB) pop-up box overlaying panels for characters, states, phenotypes and term information.

To identify the bottlenecks involved in phenotype curation (Figure 1), we estimated the time required to complete the following curation-related tasks for annotation in our manual workflow (5): (i) data preparation; (ii) annotation of characters and taxa with ontology terms; (iii) development of anatomy, taxonomy and quality ontologies and (iv) team discussions and software feedback. We documented the effort required for annotation of two datasets that were repurposed from other curation goals of the Phenoscape project. In the first dataset, which we term the full curation (FC) dataset, all of the above tasks (i–iv above) were performed for the annotation of 2699 fin, limb and girdle characters described for 2459 extant and extinct vertebrate taxa from a total of 69 publications, of which 67 were phylogenetic and two were comparative anatomical (Supplementary Table S1). The time required to complete the various curation tasks was estimated retrospectively from the work hours of a team of undergraduate students, a lead data curator, and two post-docs who were anatomy and taxonomy experts (e.g. for data preparation, we recorded the work hours for undergraduate students spent photocopying, scanning and digitizing publications and entering data using curation software). For the second dataset, termed the character annotation only (CA) dataset, only the character annotation task (ii above) was done in the context of an inter-curator annotation experiment (Manda *et al.*, in preparation). In this experiment, three curators independently annotated the same dataset for the purpose of comparison to one generated using automated annotation software. The CA dataset consisted of 203 characters randomly chosen from 7 publications of phylogenetic matrices for 499 extant and extinct vertebrate taxa that encompassed all regions of the hard and soft anatomy of vertebrates (Supplementary Table S2). Although different sets of publications were curated in the FC and CA datasets, the publications were taken from the same domain (phylogenetics of extant and extinct vertebrates), and the characters annotated were of similar complexity and format. For both FC and CA datasets, we applied the EQ formalism following the annotation guidelines for the Phenoscape research project (http://phenoscape.org/wiki/Guide_to_Character_Annotation), and curators were able to use the provisional term service within Phenex (6) (Figure 2) to create new provisional terms when required terms were missing from the ontologies. In both datasets, the same curators (a lead curator and two post-docs who were anatomy and taxonomy experts) were involved in data annotation and ontology development.

The data preparation stage for the FC dataset involved locating the literature, and as necessary, manually entering free-text characters, states, taxa and matrices. Publications were retrieved online (52 papers) or, where not electronically available, were scanned to create PDFs (17 papers). Publications dated from 1981 to 2013 and covered a wide range of fossil and extant fishes, amphibians, archosaurs and mammals (Supplementary Table S1). Where available, the associated phylogenetic matrices were downloaded from online repositories (10 matrices); otherwise the matrix data (taxonomic names, characters and states) were entered manually (59 matrices) into Mesquite (7). Files were saved in NeXML format (8) and then imported for annotation into the Phenex annotation software (4, 6).

For the FC dataset, we also estimated time spent providing feedback on software development, and time required for team discussions. Curators met for conference calls at regularly scheduled times (at least weekly) to discuss ontology term definitions, develop and improve curation standards and annotation guidelines, compare annotations to improve consistency, and work to test and improve software features. In contrast, for the CA dataset, curators were provided with a previously prepared Phenex file containing character and state descriptions, and thus were only involved in the task of character annotation. No discussion was involved in this dataset because by design each curator independently annotated the file provided to them. Therefore, we directly measured the rate of character annotation for the CA dataset. For the FC dataset, character annotation was necessarily estimated based on the average time devoted to each step in the curation workflow.

EQ annotation of characters (both datasets) and taxa (FC dataset only) with anatomy, quality and taxonomy ontologies was done using Phenex (4, 6). Anatomical entity terms were drawn from the comprehensive Uberon anatomy ontology for metazoans (9, 10), into which the amphibian (AAO) (11), teleost (TAO) (12) and vertebrate skeletal (VSAO) (13) anatomy ontologies were merged (9). Phenotypic qualities (e.g. size, shape, color) were drawn from the Phenotype and Trait (PATO) ontology (14), spatial terms were drawn from the Biological Spatial (BSPO) ontology (15), and terms for vertebrate taxa were taken from the Vertebrate Taxonomy Ontology (VTO) (16). Additions of new anatomy, taxonomy and quality classes to the ontologies were done (for FC dataset only) using the Protégé (http://protege.stanford.edu) and OBO-Edit (17) ontology editors.

## Results and discussion

Two sources of data were used to gauge the effort involved in the manual curation process, and although differences exist between the types of activities involved in generating the two datasets, comparison between rates of curation in each allows us to determine the effort for the completion of various curation tasks. The character annotation only (CA) dataset, in which three curators independently annotated a small dataset of 203 characters in the context of an annotation experiment (no data preparation, taxon annotation, ontology building or team discussions involved) resulted in an average of 583 phenotype annotations per curator, took an average of 15 h, and thus yielded an average annotation rate of 13.5 characters per hour. Annotation of the FC dataset, which included 2699 characters for 2459 vertebrate taxa, resulted in 7936 phenotypes. The completion of the FC dataset, taking data preparation, annotation of characters and taxa, ontology development, and team discussions into account, required a total of two person-years (4136 h), with a resulting overall annotation rate of 0.65 characters per hour. Considering only the character annotation component (1467 h) of the FC dataset yields an annotation rate of 1.84 characters per hour.

The large difference in character annotation rate (13.5 for the CA dataset compared with only 1.84 characters per hour for the FC dataset) is likely a result of several factors. First, although for both datasets curators used the provisional term service (6) to create new terms, curators spent more time researching term definitions in the course of curation for the FC dataset. This is because curation of the CA dataset was done in the context of an experiment, and curators were instructed to simply provide ontology term labels and parents—not definitions. Curation of the CA dataset in fact might constitute close to the ideal situation, where ontologies are fully provisioned and no new terms are required. Thus, the rate of 13.5 characters per hour could be close to the maximum amount of data that can be manually curated. Another factor contributing to the lower character annotation rate in the FC dataset is that curators were engaged in many related tasks during character annotation (e.g. discussing and updating the annotation guidelines, discussing annotation consistency and difficult examples with other curators, troubleshooting software issues). Multitasking and frequent task switching are known to reduce task efficiency (18, 19) and thus a curator’s divided attention may have contributed to the lower annotation rate. It is possible that increased specialization by curators in particular tasks, such as character annotation exclusively, may improve efficiency; however, curators necessarily engage in multiple tasks that may be more efficiently done in parallel. For example, reviewing the literature to clarify the meaning of a phenotype statement will also inform ontology development if new terms are needed to complete the annotation.

Annotation time can be reduced by incorporating Natural Language Processing (NLP) tools (20, 21) in curation software. We are actively evaluating the potential to use existing ontologies in NLP for entity markup and creation of formal EQ statements [(22), Manda *et al.*, in preparation]. This semi-automatic workflow holds potential for making the curation process more efficient, although expert review of phenotypes is still required to ensure their accurate representation. The phylogenetic systematics literature is particularly amenable to NLP because characters are by convention enumerated textually in character and state lists. However, a potentially significant start-up cost exists because much of the legacy literature is not digitized. Manual data preparation and entry constitutes 13% of the curation effort in the FC dataset. This is a significant time investment, and it is required only because most matrices (59 of 69) were not available electronically, and because even those that were available often required some manual entry of character or state descriptions. This step in the workflow could be nearly eliminated with better community data practices, in particular by encouraging authors to deposit fully populated matrices (with character and state text included in the matrix file) in public repositories in a file format that can be parsed by existing software tools, and implementing methods that ensure appropriate mark-up and deposition of phenotypic data upon publication (23–25).

In the FC dataset, ontology development required 40% of the total effort, equal to that required for annotation (Table 1). This effort included initial work on the individual vertebrate anatomy ontologies (prior to their merge into Uberon) to make terms, definitions and relations broadly applicable to the taxa under annotation (in particular, archosaurs and amphibians) and, post-merge, resulted in the addition of 243 anatomical terms (primarily fin, limb and girdle terms) to Uberon. The CA dataset also required a large number of new terms [an average of 103 anatomy terms (Supplementary Table S3) for all regions of the skeleton for the 203 characters]. The large number of new terms required for annotation of both datasets is notable given that well-developed anatomy and quality ontologies for the taxa under study (vertebrates) were used. This considerable effort, however, likely reflects the nature of character data, which contain detailed anatomical descriptions for large taxonomic groups encompassing great morphological diversity. For taxa without existing ontological representation, the effort in developing ontologies is expected to be greater.

**Table 1.** Proportion of time spent on curation tasks for the two datasets analyzed in this study (na = not applicable)

Curation tasks	FC dataset	CA only dataset
Locating literature, creating PDFs	2.9	na
Creating matrices, entering free-text taxon names, characters and character states; proofreading data	9.8	na
***Total data preparation***	***12.7***	***na***
Character annotation	35.5	100
Taxon annotation	3	na
***Total annotation work***	***38.5***	***100***
Anatomy ontology work	22.5	na
Taxonomy ontology work	16.4	na
Quality ontology work	2.6	na
***Total ontology work***	***41.5***	***na***
***Team discussions and software***	***7.3***	***na***

The first three bold, italicized rows represent categories for the total (sum) of the values of the rows above them. The last bold, italicized row is a category with only one value.

Ontology development requires intellectual effort to place a new concept in its logical context as well as the use of specialized ontology editing software. The specialized software currently required for ontology development has a steep learning curve, is prone to time-consuming errors, and is not well-suited for community editing. Although the software could be improved and simplified to make the editing process more efficient [e.g. see new tools such as TermGenie (26) and Ontology Term Organizer (OTO) (27)], the research and intellectual effort required to define anatomical and other ontology terms cannot. In our experience, the creation of well-defined anatomical terms requires consultation of the literature and researchers specializing in the anatomy of the taxa under consideration; in this case, amphibians, fishes and archosaurs. Our team included two post-docs and a data curator with expert understanding of the taxa in the literature under curation, which ensured the accurate representation of entities and phenotypes. However, outside consultation with experts was still required in some cases. For example, curators had difficulty finding a definition for a structure called ‘basilaris complex’ (28). Correspondence with experts in amphibian anatomy revealed that ‘basilaris complex’ probably referred to two adjacent structures in the amphibian inner ear. Thus, rather than defining a new single term for ‘basilaris complex’ as the curators had done provisionally, we instead created new terms for the two component structures ‘basilar papilla’ and ‘recessus basilaris’. One way of expediting ontology development is to solicit feedback from subject matter experts on a set of related terms, for example, in a workshop or data jamboree/annotation sprint setting [e.g. (13)]. Because ontologies have been applied only recently to biodiverse phenotypes, this time-consuming but essential research is required, as many new concepts need to be created in the process of annotating the comparative anatomy literature. A natural reduction in time required for anatomy ontology development can be anticipated as ontologies mature as a byproduct of continued phenotype annotation. A similar benefit may result from the development and adoption of a comprehensive global taxonomy. To the extent that community efforts are focused on development of shared resources, the ontology development load for any single research group will likely lessen over time as ontologies grow and develop through common use.

## Conclusions

Phenotype data curation is currently a time-consuming and mostly manual process that needs to be scaled up enormously to accommodate the biodiversity of life. In our work, we have found that phenotype annotation and ontology development can be equally time-consuming tasks that comprise most (80%) of the required effort. Our results suggest that phenotype annotation can be considerably streamlined if curators exclusively focus on applying entity and quality ontology terms; the feasibility of such specialized effort, however, relies on adequately developed ontologies. Further, our results show that when new terms are required, addition of provisional terms with only basic information (as in the CA dataset) as opposed to engaging in the much more time-consuming task of creating well-defined provisional terms (as in the FC dataset), streamlined the process. Subject matter experts, however, are critical to high quality ontology development. In the future, NLP tools could enable automatic markup of text with term labels, but our experience indicates that human experts will still be required to check accuracy and to appropriately add the new classes to the ontologies. Although ontology development appears to be the most non-negotiable aspect of the process, the time required for ontology development is likely to decrease naturally as the requisite ontologies are increasingly used and provisioned by the community.

## Supplementary Material

Supplementary Data
