# Supplementary material for: Moving the mountain: analysis of the effort required to transform comparative anatomy into computable anatomy
Source: Database (Oxford). 2015 May 13;2015:bav040. doi: 10.1093/database/bav040 (PMC4429748; doi:10.1093/database/bav040)
Supplement: Supplementary Data [file supp_2015_bav040_index.html]

Moving the mountain: analysis of the effort required to transform comparative anatomy into computable anatomy — Supplementary Data 

# Moving the mountain: analysis of the effort required to transform comparative anatomy into computable anatomy

## Supplementary Data

files

**Files in this Data Supplement:**

- Supplementary Data - doc file
- Supplementary Data - doc file
- Supplementary Data - doc file
