# Supplementary material for: Moving the mountain: analysis of the effort required to transform comparative anatomy into computable anatomy
Source: Database (Oxford). 2015 May 13;2015:bav040. doi: 10.1093/database/bav040 (PMC4429748; doi:10.1093/database/bav040)
Supplement: Supplementary Data [file supp_bav040_SupplementaryTable1.doc]

**Supplementary Table 1**. Annotation of the full curation (FC) dataset involved publications dating from 1981-2013 and covered a wide range of fossil and extant fishes, amphibians, archosaurs, and mammals.

| **Publication** | **Taxon** | **Annotated taxa** | **Annotated characters** | **Annotated states** | **Phenotype annotations** |
| --- | --- | --- | --- | --- | --- |
| Anderson 2001 (1) | Amphibia | 47 | 69 | 161 | 181 |
| Anderson 2007 (2) | Amphibia | 58 | 65 | 153 | 169 |
| Anderson et al. 2008 (3) | Amphibia | 53 | 36 | 81 | 98 |
| Baez and Basso 1996 (4) | Amphibia | 11 | 12 | 27 | 32 |
| Baez and Harrison 2005 (5) | Amphibia | 19 | 9 | 20 | 21 |
| Baez et al. 2009 (6) | Amphibia | 42 | 26 | 64 | 76 |
| Baldwin and Johnson 1996 (7) | Teleostei | 66 | 117 | 299 | 399 |
| Benson 2012 (8) | Synapsida | 43 | 65 | 147 | 206 |
| Brazeau 2009 (9) | Gnathostomata | 44 | 39 | 77 | 80 |
| Cannatella 1985 (10) | Amphibia | 41 | 45 | 101 | 133 |
| Carroll 2007 (11) | Amphibia | 20 | 49 | 190 | 414 |
| Chang 2004 (12) | Sarcopterygii | 13 | 24 | 51 | 61 |
| Clack et al. 2012 (13) | Tetrapoda | 18 | 20 | 45 | 74 |
| Cloutier and Arratia 2004 (14) | Actinopterygii | 42 | 103 | 234 | 251 |
| Coates and Sequiera 2001 (15) | Chondrichthyes | 18 | 32 | 60 | 74 |
| Daeschler et al. 2006 (16) | Tetrapoda | 8 | 36 | 75 | 94 |
| Davis et al. 2012 (17) | Gnathostomata | 51 | 36 | 72 | 76 |
| de Carvalho 1996 (18) | Elasmobranchii | 39 | 15 | 34 | 52 |
| Di Dario 2004 (19) | Teleostei | 112 | 156 | 358 | 480 |
| Drewes 1984 (20) | Amphibia | 54 | 9 | 22 | 36 |
| Evans et al. 2008 (21) | Amphibia | 64 | 26 | 72 | 104 |
| Fabrezi 2006 (22) | Amphibia | 62 | 26 | 70 | 92 |
| Fielitz 2004 (23) | Teleostei | 27 | 84 | 215 | 249 |
| Ford 1990 (24) | Amphibia | 41 | 45 | 105 | 146 |
| Gao and Chen 2004 (25) | Amphibia | 24 | 17 | 39 | 49 |
| Gao and Wang 2001 (26) | Amphibia | 23 | 17 | 39 | 46 |
| Gardiner et al. 2005 (27) | Actinopterygii | 14 | 6 | 12 | 15 |
| Gauthier et al. 1988 (28) | Amniota | 29 | 63 | 130 | 178 |
| Goujet 2001 (29) | Gnathostomata | 10 | 3 | 6 | 15 |
| Goujet and Young 1995 (30) | Gnathostomata | 12 | 21 | 53 | 59 |
| Hanke and Wilson 2004 (31) | Teleostomi | 19 | 27 | 61 | 66 |
| Heimberg et al. 2010 (32) | Vertebrata | 9 | 7 | 16 | 17 |
| Henrici 1998 (33) | Amphibia | 14 | 8 | 16 | 30 |
| Henrici 2009 (34) | Amphibia | 16 | 9 | 18 | 21 |
| Henrici and Haynes 2006 (35) | Amphibia | 14 | 9 | 23 | 27 |
| Heyer 1998 (36) | Amphibia | 14 | 5 | 17 | 28 |
| Holland 2013 (37) | Sarcopterygii | 19 | 15 | 31 | 42 |
| Janvier 1996 (38) | Vertebrata | 20 | 6 | 13 | 18 |
| Janvier 2007 (39) | Vertebrata | 18 | 16 | 28 | 34 |
| Johanson 2004 (40) | Sarcopterygii | 10 | 20 | 42 | 47 |
| Larson and Dimmick 1993 (41) | Amphibia | 22 | 3 | 6 | 7 |
| Lathrop 1997 (42) | Amphibia | 17 | 8 | 17 | 22 |
| Laurin 1998 (43) | Tetrapoda | 43 | 35 | 86 | 95 |
| Laurin et al. 1997 (44) | Tetrapoda | 37 | 32 | 81 | 97 |
| Lund and Grogan 2004 (45) | Chondrichthyes | 33 | 27 | 81 | 97 |
| Maisey 1981 (46) | Chordata | 53 | 62 | 123 | 154 |
| Martinez 2011 (47) | Archosauria | 20 | 76 | 159 | 195 |
| Nesbitt 2011 (48) | Archosauria | 75 | 189 | 411 | 473 |
| Nuin and do Val 2005 (49) | Amphibia | 24 | 11 | 28 | 45 |
| O’Connor and Zhou 2013 (50) | Archosauria | 58 | 162 | 380 | 469 |
| O’Leary et al. 2013 (51) | Mammalia | 83 | 63 | 134 | 141 |
| Pramuk 2006 (52) | Amphibia | 62 | 12 | 28 | 37 |
| Ruta 2003 (53) | Tetrapoda | 82 | 59 | 122 | 132 |
| Ruta 2011 (54) | Tetrapoda | 41 | 154 | 389 | 499 |
| Ruta and Bolt 2006 (55) | Amphibia | 42 | 42 | 89 | 91 |
| Ruta and Coates 2007 (56) | Amphibia | 92 | 64 | 133 | 156 |
| Scott 2005 (57) | Amphibia | 74 | 44 | 127 | 238 |
| Sereno 2009 (58) | Archosauria | 24 | 38 | 75 | 90 |
| Shirai 1996 (59) | Chondrichthyes | 37 | 20 | 51 | 74 |
| Swartz 2012 (60) | Sarcopterygii | 45 | 67 | 141 | 171 |
| Trueb and Baez 2006 (61) | Amphibia | 21 | 10 | 22 | 26 |
| Trueb et al. 2005 (62) | Amphibia | 18 | 9 | 20 | 21 |
| Vallin and Laurin 2004 (63) | Amphibia | 48 | 36 | 89 | 97 |
| Wang and Evans 2006 (64) | Amphibia | 18 | 8 | 12 | 13 |
| Wilson and Marss 2004 (65) | Vertebrata (theolodonts jawless fish) | 25 | 9 | 20 | 33 |
| Yates and Warren 2000 (66) | Amphibia | 34 | 11 | 22 | 39 |
| Zhang et al. 2009 (67) | Amphibia | 19 | 8 | 12 | 13 |
| Zhu et al.1999 (68) | Osteichthyes | 37 | 29 | 65 | 73 |
| Zhu et al. 2009 (69) | Osteichthyes | 17 | 18 | 37 | 48 |
| **Totals** |  | **2,459** | **2,699** | **6,237** | **7,936** |

**References:**

1. Anderson, J.S. (2001) The phylogenetic trunk: maximal inclusion of taxa with missing data in an analysis of the Lepospondyli (Vertebrata, Tetrapoda). *Systematic Biology*, **50**, 170-193.

2. Anderson, J.S. (2007) Incorporating ontogeny into the matrix: a phylogenetic evaluation of developmental evidence for the origin of modern amphibians. In Anderson, J.S. and Sues, H.-D. (eds.). Indiana University Press, pp. 182-227.

3. Anderson, J.S., Reisz, R.R., Scott, D.*, et al.* (2008) A stem batrachian from the Early Permian of Texas and the origin of frogs and salamanders. *Nature*, **453**, 515-518.

4. Báez, A.M., Basso, N.G. (1996) The earliest known frogs of the Jurassic of South America: review and cladistic appraisal of their relationships. In Arratia, G. (ed.). Verlag Friedrich Pfeil, Munich, Germany, pp. 131-158.

5. Báez, A.M., Harrison, T. (2005) A New Pipine Frog From An Eocene Crater Lake In North-Central Tanzania. *Palaeontology*, **48**, 723-737.

6. Báez, A.M., Moura, G.J.B., Gómez, R.O. (2009) Anurans from the Lower Cretaceous Crato Formation of northeastern Brazil: implications for the early divergence of neobatrachians. *Cretaceous Research*, **30**, 829-846.

7. Baldwin, C.C., Johnson, G.D. (1996) Interrelationships of Aulopiformes. In Stiassny, M.L.J., Parenti, L.R. and Johnson, G.D. (eds.), pp. 355-404.

8. Benson, R.B.J. (2012) Interrelationships of basal synapsids: cranial and postcranial morphological partitions suggest different topologies. *Journal of Systematic Palaeontology*, **10**, 601-624.

9. Brazeau, M.D. (2009) The braincase and jaws of a Devonian 'acanthodian' and modern gnathostome origins. *Nature*, **457**, 305-308.

10. Cannatella, D.C. (1985) A phylogeny of primitive frogs (archaeobatrachians). University of Kansas.

11. Carroll, R.L. (2007) The Palaeozoic Ancestry of Salamanders, Frogs and Caecilians. *Zoological Journal of the Linnean Society*, **150**, 1-140.

12. Chang, M.-M. (2004) Synapomorphies and scenarios - more characters of Youngolepis betraying its affinity to the Dipnoi. In Arratia, G., Wilson, M.V.H. and Cloutier, R. (eds.), pp. 665-686.

13. Clack, J.a., Ahlberg, P.E., Blom, H.*, et al.* (2012) A new genus of Devonian tetrapod from North-East Greenland, with new information on the lower jaw of Ichthyostega. *Palaeontology*, **55**, 73-86.

14. Cloutier, R., Arratia, G. (2004) Early diversification of actinopterygians. In Arratia, G., Wilson, M. and Cloutier, R. (eds.). Verlag Dr. Friedrich Pfeil München, pp. 217-270.

15. Coates, M.I., Sequeira, S.E.K. (2001) A new stethacanthid chondrichthyan from the lower Carboniferous of Bearsden, Scotland. *Journal of Vertebrate Paleontology*, **21**, 438-459.

16. Daeschler, E.B., Shubin, N.H., Jenkins, F.A., Jr. (2006) A Devonian tetrapod-like fish and the evolution of the tetrapod body plan. *Nature*, **440**, 757-763.

17. Davis, S.P., Finarelli, J.a., Coates, M.I. (2012) Acanthodes and shark-like conditions in the last common ancestor of modern gnathostomes. *Nature*, **486**, 247-250.

18. de Carvalho, M. (1996) Higher-Level Elasmobranch Phylogeny, Basal Squaleans, and Paraphyly. pp. 35-62.

19. Di Dario, F. (2004) Relações filogenéticas entre os grandes grupos de Clupeomorpha e suas possíveis relações com Ostariophysi (Actinopterygii, Teleostei).

20. Drewes, R.C. (1984) A phylogenetic analysis of the Hyperoliidae (Anura): treefrogs of Africa, Madagascar, and the Seychelles Islands. *Occasional Papers of the California Academy of Sciences*, **139**, 1-70.

21. Evans, S.E., Jones, M.E.H., Krause, D.W. (2008) A giant frog with South American affinities from the Late Cretaceous of Madagascar. *Proceedings of the National Academy of Sciences USA*, **105**, 2951-2956.

22. Fabrezi, M. (2006) Morphological evolution of the Ceratophryinae (Anura, Neobatrachia). *Journal of Zoological Systematics and Evolutionary Research*, **44**, 153-166.

23. Fielitz, C. (2004) The phylogenetic relationships of the Enchodontidae (Teleostei: Aulopiformes). In Arratia, G., Wilson, M. and Cloutier, R. (eds.), pp. 619-634.

24. Ford, L.S. (1990) The phylogenetic position of poison-dart frogs (Dendrobatidae): reassessment of the neobatrachian phylogeny with commentary on complex character systems. pp. 307-307.

25. Gao, K.-Q., Chen, S. (2004) A new frog (Amphibia: Anura) from the Lower Cretaceous of western Liaoning, China. *Cretaceous Research*, **25**, 761-769.

26. Gao, K.-Q., Wang, Y. (2001) Mesozoic anurans from the Liaoning Province, China, and phylogenetic relationships of archaeobatrachian anuran clades. *Journal of Vertebrate Paleontology*, **21**, 460-476.

27. Gardiner, B.G., Schaeffer, B., Masserie, J.A. (2005) A review of the lower actinopterygian phylogeny. *Zoological Journal of the Linnean Society*, **144**, 511-525.

28. Gauthier, J.A., Kluge, A.G., Rowe, T. (1988) Amniote phylogeny and the importance of fossils. *Cladistics*, **4**, 105-209.

29. Goujet, D. (2001) Placoderms and basal gnathostome apomorphies. pp. 209-222.

30. Goujet, D., Young, G.C. (1995) Interrelationships of placoderms revisited. *Geobios, Mem. Spec.*, **19**, 89-95.

31. Hanke, G.F., Wilson, M.V.H. (2004) New teleostome fishes and acanthodian systematics. In Arratia, G., Wilson, M. and Cloutier, R. (eds.), Munchen, Germany, pp. 189-216.

32. Heimberg, A.M., Cowper-Sal-lari, R., Sémon, M.*, et al.* (2010) microRNAs reveal the interrelationships of hagfish, lampreys, and gnathostomes and the nature of the ancestral vertebrate. *Proceedings of the National Academy of Sciences of the United States of America*, **107**, 19379-19383.

33. Henrici, A.C. (1998) A new pipoid anuran from the Late Jurassic Morrison Formation at Dinosaur National Monument, Utah. *Journal of Vertebrate Paleontology*, **18**, 321-332.

34. Henrici, A.C. (2009) Reassessment of Scaphiopus neuter Kluge, 1966 (Anura: Pelobatoidea: Pelobatidae), based on new material from Anceney, Montana (Early Barstovian). *Annals of the Carnegie Museum*, **78**, 273-287.

35. Henrici, A.C., Haynes, S.R. (2006) Elkobatrachus brocki, a new pelobatid (Amphibia: Anura) from the Eocene Elko Formation of Nevada. *Annals of the Carnegie Museum*, **75**, 11-35.

36. Heyer, W.R. (1998) The relationships of Leptodactylus diedrus (Anura, Leptodactylidae). *Alytes*, **16**, 1-24.

37. Holland, T. (2013) Pectoral girdle and fin anatomy of Gogonasus andrewsae long, 1985: Implications for tetrapodomorph limb evolution. *Journal of morphology*, **274**, 147-164.

38. Janvier, P. (1996) The dawn of the vertebrates: characters versus common ascent in the rise of current vertebrate phylogenies. *Palaeontology*, **39**, 259-287.

39. Janvier, P. (2007) Homologies and evolutionary transitions in early vertebrate history. In Anderson, J.S. and Sues, H.-D. (eds.). Indiana University Press, Bloomington, pp. 57-121.

40. Johanson, Z. (2004) Late Devonian sarcopterygian fishes from eastern Gondwana (Australia and Antarctica) and their importance in phylogeny and biogeography. In Arratia, G., Wilson, M. and Cloutier, R. (eds.). Verlag Dr. Friedrich Pfeil, Munchen, pp. 287-308.

41. Larson, A., Dimmick, W.W. (1993) Phylogenetic relationships of the salamander families: an analysis of congruence among morphological and molecular characters. *Herpetological Monographs*, **7**, 77-93.

42. Lathrop, A. (1997) Taxonomic review of the megophryid frogs (Anura: Pelobatoidea). *Asiatic Herpetological Research*, **7**, 68-79.

43. Laurin, M. (1998) The importance of global parsimony and historical bias in understanding tetrapod evolution. Part I. Systematics, middle ear evolution and jaw suspension. *Annales des Sciences Naturelles*, **19**, 1-42.

44. Laurin, M., Reisz, R.R., Sumida, S.S.*, et al.* (1997) A new perspective on tetrapod phylogeny. *Amniote origins: completing the transition to land*.

45. Lund, R., Grogan, E.D. (2004) Five new euchondrocephalan Chondrichthyes from the Bear Gulch Limestone (Serpukhovian, Namurian E2b) of Montana, USA. In Arratia, G., Wilson, M. and Cloutier, R. (eds.), pp. 505-531.

46. Maisey, J.G. (1981) Heads and Tails: A Chordate Phylogeny. *Cladistics*, **2**, 201-256.

47. Martinez, R.N., Sereno, P.C., Alcober, O.a.*, et al.* (2011) A basal dinosaur from the dawn of the dinosaur era in southwestern Pangaea. *Science*, **331**, 206-210.

48. Nesbitt, S.J. (2011) The Early Evolution of Archosaurs: Relationships and the Origin of Major Clades. *Bulletin of the American Museum of Natural History*, 1-292.

49. Nuin, P.A.S., do Val, F.C. (2005) Phylogenetic analysis of the subfamily Hylodinae (Anura, Leptodactylidae) based on morphological characters. *Amphibia-Reptilia*, **26**, 139-147.

50. O’Connor, J.K., Zhou, Z. (2013) A redescription of Chaoyangia beishanensis (Aves) and a comprehensive phylogeny of Mesozoic birds. *Journal of Systematic Palaeontology*, **11**, 889-906.

51. O'Leary, M.a., Bloch, J.I., Flynn, J.J.*, et al.* (2013) The placental mammal ancestor and the post-K-Pg radiation of placentals. *Science*, **339**, 662-667.

52. Pramuk, J.B. (2006) Phylogeny of South American Bufo (Anura: Bufonidae) inferred from combined evidence. *Zoological Journal of the Linnean Society*, **146**, 407-452.

53. Ruta, M., Coates, M.I., Quicke, D.L.J. (2003) Early tetrapod relationships revisited. *Biological Reviews*, **78**, 251-345.

54. Ruta, M. (2011) Phylogenetic signal and character compatibility in the appendicular skeleton of early tetrapods. *Special papers in Palaeontology*, **86**, 31-43.

55. Ruta, M., Bolt, J.R. (2006) A reassessment of the temnospondyl amphibian Perryella olsoni from the Lower Permian of Oklahoma. *Transactions of the Royal Society of Edinburgh: Earth Sciences*, **97**, 113-165.

56. Ruta, M., Coates, M.I. (2007) Dates, nodes and character conflict: Addressing the Lissamphibian origin problem. *Journal of Systematic Palaeontology*, **5**, 69-122.

57. Scott, E. (2005) A phylogeny of ranid frogs (Anura: Ranoidea: Ranidae), based on a simultaneous analysis of morphological and molecular data. *Cladistics*, **21**, 507-574.

58. Sereno, P.C., Tan, L., Brusatte, S.L.*, et al.* (2009) Tyrannosaurid skeletal design first evolved at small body size. *Science*, **326**, 418-422.

59. Shirai, S. (1996) Phylogenetic interrelationships of neoselachians (Chondrichthyes: Euselachii). In Stiassny, M.L.J., Parenti, L.R. and Johnson, G.D. (eds.). Academic Press, pp. 9-34.

60. Swartz, B. (2012) A marine stem-tetrapod from the Devonian of western North America. *PloS one*, **7**, e33683-e33683.

61. Trueb, L., Báez, A.M. (2006) Revision of the Early Cretaceous Cordicephalus from Israel and an assessment of its relationships among pipoid frogs. *Journal of Vertebrate Paleontology*, **2006**, 44-59.

62. Trueb, L., Ross, C.F., Smith, R. (2005) A new pipoid anuran from the Late Cretaceous of South Africa. *Journal of Vertebrate Paleontology*, **25**, 533-547.

63. Vallin, G., Laurin, M. (2004) Cranial morphology and affinities of Microbrachis, and a reappraisal of the phylogeny and lifestyle of the first amphibians. *Journal of Vertebrate Paleontology*, **24**, 56-72.

64. Wang, Y., Evans, S.E. (2006) A new short-bodied salamander from the Upper Jurassic/Lower Cretaceous of China. *Acta Palaeontologica Polonica*, **51**, 127-130.

65. Wilson, M.V.H., Marss, T. (2004) Toward a phylogeny of the thelodonts. In Arratia, G., Wilson, M. and Cloutier, R. (eds.), pp. 95-108.

66. Yates, A.M., Warren, A.A. (2000) The phylogeny of the ‘higher’ temnospondyls (Vertebrata: Choanata) and its implications for the monophyly and origins of the Stereospondyli. *Zoological Journal of the Linnean Society*, **128**, 77-121.

67. Zhang, G., Wang, Y., Jones, M.E.H.*, et al.* (2009) A new Early Cretaceous salamander (Regalerpeton weichangensis gen. et sp. nov.) from the Huajiying Formation of northeastern China. *Cretaceous Research*, **330**, 551-558.

68. Zhu, M., Yu, X., Janvier, P. (1999) A primitive fossil fish sheds light on the origin of bony fishes. *Nature*, **397**, 607-610.

69. Zhu, M., Zhao, W., Jia, L.*, et al.* (2009) The oldest articulated osteichthyan reveals mosaic gnathostome characters. *Nature*, **458**, 469-474.
