# Supplementary material for: Moving the mountain: analysis of the effort required to transform comparative anatomy into computable anatomy
Source: Database (Oxford). 2015 May 13;2015:bav040. doi: 10.1093/database/bav040 (PMC4429748; doi:10.1093/database/bav040)
Supplement: Supplementary Data [file supp_bav040_SupplementaryTable2.doc]

# Supplementary Table 2. The character annotation only (CA) dataset involved publications dated from 2005-2013 and covered a wide range of fossil and extant fishes, amphibians, archosaurs, and mammals.

| **Author** | **Taxon** | **Characters annotated** | **Character states annotated** | **Average number of Phenotypes** |
| --- | --- | --- | --- | --- |
| Chakrabarty 2007 (1) | Fishes (cichlids) | 29 | 61 | 72 |
| Coates and Sequiera 2001 (2) | Chondrichthyes | 29 | 58 | 70 |
| Conrad 2008 (3) | Squamate reptiles (iguanids and snakes) | 29 | 74 | 98 |
| Hill 2005 (4) | Amniotes | 29 | 75 | 98 |
| Nesbitt et al. 2011 (5) | Birds | 29 | 59 | 83 |
| O’Leary et al. 2013 (6) | Mammals | 29 | 63 | 70 |
| Skutschas and Gubin 2012 (7) | Amphibians | 29 | 70 | 92 |
| **Totals** |  | **203** | **460** | **583** |

**References:**

1. Chakrabarty, P. (2007) A morphological phylogenetic analysis of middle american cichlids with special emphasis on the section '*Nandopsis*' *sensu* Regan. *Miscellaneous Publications Museum of Zoology University of Michigan*, **198**, 1-31.

2. Coates, M.I. and Sequeira, S.E.K. (2001) A new stethacanthid chondrichthyan from the lower Carboniferous of Bearsden, Scotland. *Journal of Vertebrate Paleontology*, **21**, 438-459.

3. Conrad, J.L. (2008) Phylogeny And Systematics Of Squamata (Reptilia) Based On Morphology. *Bulletin of the American Museum of Natural History*, **310**, 1-182.

4. Hill, R.V. (2005) Integration of Morphological Data Sets for Phylogenetic Analysis of Amniota: The Importance of Integumentary Characters and Increased Taxonomic Sampling. *Systematic Biology*, **54**, 530-547.

5. Nesbitt, S.J. (2011) The Early Evolution of Archosaurs: Relationships and the Origin of Major Clades. *Bulletin of the American Museum of Natural History*, 1-292.

6. O'Leary, M.a., Bloch, J.I., Flynn, J.J., Gaudin, T.J., Giallombardo, A., Giannini, N.P., Goldberg, S.L., Kraatz, B.P., Luo, Z.-X., Meng, J. *et al.* (2013) The placental mammal ancestor and the post-K-Pg radiation of placentals. *Science*, **339**, 662-667.

7. Skutschas, P.P. and Gubin, Y.M. (2012) A New Salamander from the Late Paleocene—Early Eocene of Ukraine. *Acta Palaeontologica Polonica*, **57**, 135-148.
