# Supplementary material for: Moving the mountain: analysis of the effort required to transform comparative anatomy into computable anatomy
Source: Database (Oxford). 2015 May 13;2015:bav040. doi: 10.1093/database/bav040 (PMC4429748; doi:10.1093/database/bav040)
Supplement: Supplementary Data [file supp_bav040_SupplementaryTable3.doc]

**Supplementary Table 3.** The number of provisional terms created by individual curators and time required to complete curation of the 203 characters in the CA dataset (“knowledge round” of the inter-curator annotation experiment described in Manda et al., in prep).

| **Curator** | **Uberon** | **PATO** | **BSPO** | **Curation time (hours)** |
| --- | --- | --- | --- | --- |
| Curator 1 | 129 | 74 | 3 | 15 |
| Curator 2 | 72 | 52 | 0 | 15 |
| Curator 3 | 108 | 35 | 3 | 14 |
| Average | 103 | 54 | 2 | 14.7 |
